# The legality and appropriateness of keeping Korean Medical Licensing Examination items confidential: a comparative analysis and review of court rulings

**DOI:** 10.3352/jeehp.2024.21.28

**Published:** 2024-10-15

**Authors:** Jae Sun Kim, Dae Un Hong, Ju Yoen Lee

**Affiliations:** 1 College of Law, Dongguk University, Seoul, Korea; 2 School of Law, Hanyang University, Seoul, Korea; Hallym University, Korea

**Keywords:** Confidentiality, Disclosure, Health personnel, Medical licensure, Republic of Korea

## Abstract

This study examines the legality and appropriateness of keeping the multiple-choice question items of the Korean Medical Licensing Examination (KMLE) confidential. Through an analysis of cases from the United States, Canada, and Australia, where medical licensing exams are conducted using item banks and computer-based testing, we found that exam items are kept confidential to ensure fairness and prevent cheating. In Korea, the Korea Health Personnel Licensing Examination Institute (KHPLEI) has been disclosing KMLE questions despite concerns over exam integrity. Korean courts have consistently ruled that multiple-choice question items prepared by public institutions are non-public information under Article 9(1)(v) of the Korea Official Information Disclosure Act (KOIDA), which exempts disclosure if it significantly hinders the fairness of exams or research and development. The Constitutional Court of Korea has upheld this provision. Given the time and cost involved in developing high-quality items and the need to accurately assess examinees’ abilities, there are compelling reasons to keep KMLE items confidential. As a public institution responsible for selecting qualified medical practitioners, KHPLEI should establish its disclosure policy based on a balanced assessment of public interest, without influence from specific groups. We conclude that KMLE questions qualify as non-public information under KOIDA, and KHPLEI may choose to maintain their confidentiality to ensure exam fairness and efficiency.

## Graphical abstract

[Fig f1-jeehp-21-28]

## Introduction

### Background

The Korea Health Personnel Licensing Examination Institute (KHPLEI) has prepared multiple-choice question (MCQ) items for the Korean Medical Licensing Examination (KMLE) utilizing an item bank. In the past, the MCQ items used in each KMLE were not made public, and Korean courts ruled that they were not subject to disclosure, supporting KHPLEI’s non-disclosure policy [1]. However, after subsequent controversy, KHPLEI voluntarily disclosed the MCQ items used in the KMLE [2,3]. Nevertheless, the question of whether KMLE items should be made public remains a sensitive issue within the medical community.

For example, in 2017, a bill was proposed to require the disclosure of items for licensing exams for healthcare professionals [4]; however, when the Korean Medical Association (KMA) expressed its opposition [5], the Korean Medical Student Association criticized the KMA [6]. Disagreement on this issue exists not only among interest groups such as medical students [7], medical professors, the KMA, and KHPLEI, but also among members within each group [8].

In 2022, KHPLEI introduced computer-based testing (CBT) for the KMLE, replacing paper-based testing [9]. Additionally, KHPLEI is considering the introduction of computerized adaptive testing (CAT), in which items are adjusted based on the examinee’s answers [10-12]. The advantages of the CAT method in medical licensing examinations have already been reported [13], and recent studies have reaffirmed this point [14,15]. The implementation of the CAT format is feasible only under the CBT examination method, which generates items from an item bank. However, KHPLEI argues that disclosing KMLE items presents a significant obstacle to the operation of its item bank and the CBT method [16]. Furthermore, research suggests that maintaining a policy of non-disclosure for the actual KMLE items is desirable [8].

### Objectives

This study examined whether it is appropriate to keep the items of the KMLE confidential by conducting a case study of countries that use the CBT method and an item bank for their medical licensing examinations. It also investigates whether it is legal under Korean law for the KHPLEI to withhold the KMLE’s items.

## Methods

### Ethics statement

This article presents a study of legal policies based on legislation and judicial precedents. As it did not involve research with human subjects, Institutional Review Board approval was not required.

### Study design

This was a review based on literature, legislative texts, and judicial precedents.

### Information sources and search strategy

First, to determine whether it is appropriate for the KHPLEI to keep the items of the KMLE confidential, we studied and analyzed the cases of countries that, like Korea, prepare exam items utilizing an item bank and administer their medical licensing examinations in CBT format. Next, to examine the legality of keeping the MCQ items of the KMLE confidential, we identified relevant legal issues and analyzed Korean court judgments regarding whether items prepared by public institutions, including the KMLE, should be disclosed or kept confidential.

Information on the court cases cited in this article was obtained from the following free and commercial databases, including internet materials:

For U.S. court opinions:

• Thomson Reuters, Westlaw Precision: https://1.next.westlaw.com

For Korean court judgments:

• Court of Korea, Comprehensive Legal Information Database: https://glaw.scourt.go.kr/wsjo/intesrch/sjo022.do

• Court of Korea, Court Electronic Records: https://www.scourt.go.kr/portal/information/events/search/search.jsp

• CaseNote: https://casenote.kr/

• Legal Engine: https://legalengine.co.kr/

For Korean court judgments not found in the aforementioned legal databases, copies can be accessed through a request to the courts of Korea [17].

## Results

### United States

The National Board of Medical Examiners (NBME) administers the United States Medical Licensing Examination (USMLE). Since 1999, the USMLE has been conducted in a CBT format, after discontinuation of the older paper-based exam [18,19]. For security and reusability reasons, the NBME does not disclose the exam items used in each USMLE [20]. Instead, it provides sample items to help medical students become familiar with the exam format [21]. Examinees must keep the exam items they encounter in the USMLE confidential, and it is illegal for any test prep service or program to provide those exam items. If an examinee uses such a service or program, their exam score may be canceled, thereby invalidating their results even if they receive a passing score [22].

In *NBME v. Optima* (2011) [23], the NBME brought a copyright infringement action against Optima, a company that offered USMLE test preparation courses. Optima had induced multiple individuals to take USMLE exams to copy test items and forward them to Optima for use in its course materials. Consequently, the court held that Optima willfully infringed upon the NBME’s copyrighted USMLE items and awarded the NBME maximum statutory damages and injunctive relief. In *Thomas v. NBME* (2015) [24], a former Optima enrollee sued the NBME after it invalidated his passing score on the USMLE, but the court ruled in favor of the NBME. Recently, the NBME canceled the passing scores of examinees suspected of sharing or accessing secure exam content prior to testing. In *Giri v. NBME* (2024) [25], the court upheld the NBME’s actions.

### Canada and Australia

The situations in Canada and Australia are similar to that in the United States. Like the USMLE, the Medical Council of Canada Qualifying Examination Part I and the Australian Medical Council CAT MCQ examination are computer-based exams [26,27]. These exam materials are protected by copyright and must be kept confidential [28,29]. For instance, examinees are prohibited from discussing, copying, reproducing, or sharing examination content in any form, including from memory [29,30]. Instead, the Medical Council of Canada and the Australian Medical Council provide basic information, including sample questions, to help examinees familiarize themselves with the types of questions they will encounter on the exams [31,32].

### South Korea

#### Relevant legislation and legal issues

The right to know is recognized as a constitutional right, despite the absence of an explicit provision for it [33,34]. The Korean Official Information Disclosure Act (KOIDA) regulates the disclosure of information by public institutions to ensure the people’s right to know (Article 1 of KOIDA) [35]. According to the Act, information held by public institutions is generally subject to disclosure, and institutions are required to disclose such information (Articles 3 and 9(1) of KOIDA) [35]. However, the Act also lists confidential information that public institutions may choose not to disclose, including “information concerning... tests... which, if disclosed, is reasonably likely to cause significant disruption to the fairness of work or the performance of research and development” (Article 9(1)(v) of KOIDA) [35].

KHPLEI, which administers the KMLE, is a special legal entity established under the Korea Health Personnel Licensing Examination Institute Act [36] and is classified as a public institution subject to KOIDA (Article 2(3)(v) of the Act and Article 2(4) of its Enforcement Decree) [35,37]. Therefore, KHPLEI is generally required to disclose the information it holds and manages under KOIDA. The question arises as to whether KMLE items prepared by KHPLEI are subject to disclosure or if they qualify as “information concerning tests” and “information that, if disclosed, is likely to cause significant disruption to the fairness of the work or the performance of research and development,” thereby exempting them from disclosure.

#### 1990s court rulings

In 1999, the Seoul Administrative Court ruled that the MCQ items for the first round of the Korean Judicial Examination, which were prepared using an item bank, were not subject to disclosure (Table 1). The court opined: “If the test questions and answers are disclosed, it will be impossible to reuse identical or similar items, and the number of items in an item bank is necessarily limited for the Korean Judicial Examination, which is designed for individuals who have completed advanced studies in a specialized subject. This limitation would ultimately hinder the normal maintenance of the item bank after several years…” Additionally, the court noted, “There is substantial reason to believe that disclosing the test would cause significant disruption to the fairness of the work or the performance of research and development” [38]. This decision was upheld by the Seoul High Court and later by the Supreme Court of Korea [39,40]. In the same year, the Seoul Administrative Court ruled that the multiple-choice test questions for the Certified Professional Logistician Examination were also information not subject to disclosure for the same reasons [41].

#### 2000s court rulings

In the 2000s, there were 2 lawsuits aimed at overturning KHPLEI’s decision to refuse to disclose the MCQ items for the Korean Dental Licensing Examination (KDLE) (Table 2). In the first lawsuit (2003), the court sided with KHPLEI, ruling that the exam items were non-public information, as it had done in the Korean Judicial Examination case involving the Ministry of the Interior [42-44]. Specifically, the Seoul High Court reasoned [43]:

“Since the test items are prepared utilizing an item bank, releasing them would make it virtually impossible to use questions identical or similar to previous ones. If questions used in each KDLE are released over several years, maintaining the item bank would become increasingly difficult. Even if new questions are developed annually, the range of potential questions will gradually narrow, complicating the question creation process. Additionally, accurately measuring a test taker’s ability would become more challenging. Consequently, even test takers with no knowledge of the subject could pass by merely memorizing past exam questions and answers, undermining the test’s function as an evaluation tool and raising concerns about the quality of those who obtain a license.”

In the case seeking disclosure of the MCQ items from the 2004 KMLE, the plaintiff was a test taker who failed the exam by 1.5 points. In the first instance, the first instance sided with the plaintiff, ruling that the questions and answers did not constitute non-public information [45]. However, the appellate court reversed this decision, stating that “if past items of the KMLE selected from an item bank are disclosed, it will be impossible to use identical or similar questions again, ultimately jeopardizing the normal operation of the question bank.” The court also expressed concern that disclosing items used in a test would hinder the accurate assessment of exam takers’ abilities [1].

Despite the court’s ruling in the Korean Judicial Examination case, the Ministry of the Interior had been releasing the exam items used in each first round of the Korean Judicial Examination since 2001. This practice influenced the second KDLE case (2006–2007), where the Seoul High Court ruled in favor of the plaintiff, overturning the Seoul Administrative Court’s decision to support KHPLEI [46,47]. The Seoul High Court stated that there was no reason to treat the KDLE and the Korean Judicial Examination differently [47]. However, the Supreme Court of Korea reversed the decision and remanded the case to the Seoul High Court. The court noted that the Korean Judicial Examination and the KDLE are not necessarily comparable, opining that whether the disclosure of past exam items would substantially interfere with the fair performance of duties should be determined on an individual basis, considering the nature and content of the particular examination, the specifics of the disclosure, and the potential increase in workloads and spillover effects [48]. The court added, “Given that utilizing the item bank offers many advantages, such as ensuring high-quality items while reducing the time and cost of preparation, there is substantial reason to believe that disclosing the examination items would significantly hinder the fair performance of examination administration or research and development. Therefore, KHPLEI may choose not to disclose the items [48].” Subsequently, the Seoul High Court changed its former stance, ruling that the items used in the KDLE were confidential information [49]. When the plaintiff appealed again, the Supreme Court of Korea affirmed this decision of the Seoul High Court [50].

Meanwhile, the plaintiff of the second KDLE case filed a constitutional complaint claiming that Article 9(1)(v) of the KOIDA, which served as the legal basis for KHPLEI to keep the exam questions confidential, violated people’s right to know. In response, the Constitutional Court upheld this provision constitutional, stating that “the public interest in ensuring the fairness and accuracy of examinations conducted by public institutions, as supported by Article 9(1)(v) of the KOIDA, cannot be considered inferior to the plaintiff’s right to know [51].”

In 2008, a case was brought seeking the disclosure of the items used in the Korean Oriental Medical Licensing Examination (KOMLE). The court ruled that the exam items were not subject to disclosure, applying the same reasoning used in the rulings on the previous KMLE and KDLE cases [52-54]. In addition, the plaintiff of the KOMLE case challenged the constitutionality of Article 9(1)(v) of the KOIDA and the Constitutional Court of Korea ruled, as it had before, that this provision was not unconstitutional [55].

#### 2010s court rulings

Publishers began to sell collections of past items from the KMLE and the Korean Nursing Licensing Examination. They asked exam takers to recall the exam items and paid them for the items they provided. In response, the prosecution charged these publishers with copyright infringement. In 2012, the court recognized the copyrightability of the items and found these publishers guilty [56].

Another lawsuit was filed seeking the disclosure of the exam items used in the KDLE, but the court upheld its previous position that the exam items were not subject to disclosure [57-59]. Specifically, the Seoul High Court stated that “there are no new circumstances that require a change in the previous decision of the Supreme Court of Korea [48], which stated that KDLE exam items are not subject to disclosure under Article 9(1)(v) of KOIDA” (Table 3) [58].

Although it did not pertain to MCQ items, a lawsuit was filed in 2018 seeking the disclosure of information regarding the clinical skills examination conducted during the 2018 KMLE. Examinees are randomly assigned to take 6 out of 54 clinical performance examinations (CPXs) and 6 out of 32 objective structured clinical examinations (OSCEs) in the KMLE clinical skills examination. Although the 54 CPXs and the 32 OSCEs have already been disclosed, the plaintiff sought additional information, including the checklist for each of the 32 OSCEs. The court ruled that disclosing specifics of the checklist would enable examinees to prepare in advance based on that information, thereby making it difficult to accurately assess their overall clinical skills. Therefore, this information was determined to be non-public [60] (Table 4).

#### 2020s Court rulings

Lawsuits seeking the disclosure of multiple-choice test questions prepared by public institutions continued into the 2020s. In response, Korean courts have reaffirmed their stance that these exam questions are non-public information, as indicated in Table 5 [61-63].

## Discussion

### Key results

This study examined cases from the United States, Canada, and Australia, which use question banks to create medical licensing exam items and utilize the CBT format. In these countries, all agencies responsible for administering medical licensing exams keep the actual exam items confidential. Not only is copying exam items at the test site prohibited, but examinees are also forbidden from recalling items from memory and sharing them with others. Those who illegally access leaked exam items in advance and then take the exam are treated as cheaters, resulting in the cancellation of their exam scores to ensure the fairness of the examination.

### Interpretation

According to Article 9(1)(v) of the KOIDA, upheld as constitutional by the Constitutional Court of Korea [51,55], if there are reasonable grounds to believe that the disclosure of examination items prepared by public institutions would significantly hinder the fair performance of examination administration or the research and development of examination items, those items may be classified as non-public information. Based on this provision, Korean courts have ruled that MCQ items prepared by public institutions from item banks are non-public information, and this stance has been firmly established in Korean jurisprudence. These well-established court rulings apply to the KMLE administered by KHPLEI.

### Implications

The KHPLEI is currently releasing the MCQ items used in the KMLE. Since Article 9(1)(v) of the KOIDA provides public institutions with the option, rather than the obligation, to withhold non-public information, KHPLEI has the discretion to disclose the exam questions. However, if KHPLEI’s decision to disclose the exam items is made in response to demands from medical students or pressure from political groups, rather than being based on its own proper and reasonable judgment, this approach could be problematic. As a special public institution administering Korean health personnel licensing examinations, including the KMLE, KHPLEI should establish its disclosure policy with consideration for the fairness and efficiency of the examination aimed at selecting qualified medical practitioners. Given that KHPLEI’s policy will ultimately impact public health, it must not be influenced by the interests of any specific group, but rather reflect an objective assessment of the situation from the perspective of the general public.

As the U.S. Court recognized in *NBME v. Optima* [23], developing MCQ items for the medical licensing exam is both time-consuming and expensive. If these MCQs are made public, it will necessitate abandoning the reuse of proven high-quality questions, making it increasingly difficult to prepare exam questions. Additionally, test takers may focus their preparation on frequently appearing items, which could significantly hinder the accurate measurement of their abilities. For this reason, the Korean courts and the Constitutional Court have upheld the non-disclosure of MCQ items used in health personnel licensing examinations such as the KMLE, KDLE, and KOMLE [1,43,48,51,53,55,58]. While the KHPLEI currently discloses past MCQ items of the KMLE, this practice does not change the nature of the questions from non-public information to public information. Instead, the ongoing calls for the confidentiality of MCQ items within KHPLEI [16] indicate that the negative effects of disclosure remain a significant concern. In particular, if the examination is administered in the CAT format in the future, the need for confidentiality will be even greater.

## Conclusion

The MCQ items used in the KMLE need to be kept confidential, and these questions are classified as non-public information under Article 9(1)(v) of the KOIDA. Therefore, the KHPLEI may, based on its proper and reasonable judgment, choose to discontinue the disclosure of the MCQ items and maintain their confidentiality.

## Figures and Tables

**Figure f1-jeehp-21-28:**
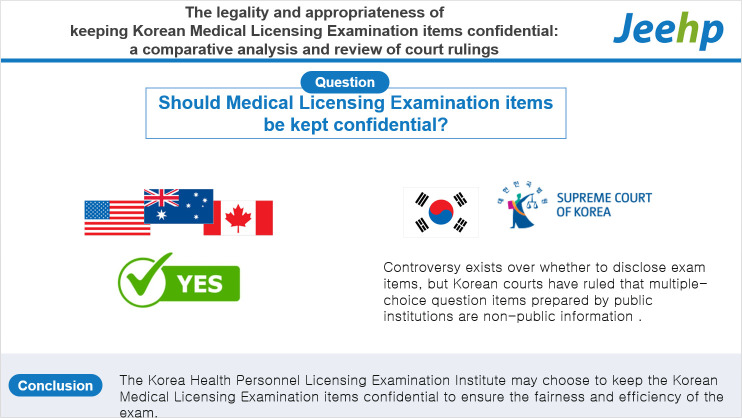


**Table 1.** Court cases from the 1990s regarding multiple-choice question items prepared by public institutions

Subject of disclosure request	Defendant	Court procedural history	
		Court name & case no. (decision date)	Court’s decision
Exam questions for the first round of the Korean Judicial Examination	Minister of the Interior	Seoul Administrative Court 99Gu2788, 14194 Judgment (Jul 2, 1999)	Non-public information
		Seoul High Court 99Nu9232 Judgment (Nov 24, 1999)	Non-public information
		Supreme Court of Korea 99Du12205 Judgment (Feb 23, 2000)	Affirm
Certified Professional Logistician Examination questions	Minister of Infrastructure and Transport	Seoul Administrative Court 99Gu7004 Judgment (Oct 15, 1999)	Non-public information (affirmed without appeal)

**Table 2.** Court cases from the 2000s regarding multiple-choice question items of Korean health personnel licensing examinations

Subject of disclosure request	Defendant	Court procedural history	
		Court name & case no. (decision date)	Court’s decision
Korean Dental Licensing Examination questions	KHPLEI	Seoul Administrative Court 2003Gu-hap13830 Judgment (Sep 3, 2003)	Non-public information
		Seoul High Court 2003Nu17155 Judgment (Jul 22, 2004)	Non-public information
		Supreme Court of Korea 2004Du9890 Judgment (Dec 10, 2004)	Affirm
Korean Medical Licensing Examination questions	KHPLEI	Seoul Administrative Court 2004Gu-hap8071 Judgment (Jul 27, 2004)	Public information
		Seoul High Court 2004Nu17145 Judgment (Sep 2, 2005)	Non-public information (affirmed without appeal)
Korean Dental Licensing Examination questions	KHPLEI	Seoul Administrative Court 2005Gu-hap18570 Judgment (Jan 6, 2006)	Non-public information
		Seoul High Court 2006Nu3522 Judgment (Sep 22, 2006)	Public information
		Supreme Court of Korea 2006Du15936 Judgment (Jun 15, 2007)	Non-public information
		Seoul High Court 2007Nu15805 Judgment (Sep 14, 2007)	Non-public information
		Supreme Court of Korea 2007Du21723 Judgment (Dec 27, 2007)	Affirm
Korean Oriental Medicine Licensing Examination	KHPLEI	Seoul Administrative Court 2008Gu-hap15534 Judgment (Jul 30, 2008)	Non-public information
		Seoul High Court 2008Nu22947 Judgment (Jun 24, 2010)	Non-public information
		Supreme Court of Korea 2010Du12927 Judgment (Sep 9, 2010)	Affirm

KHPLEI, Korea Health Personnel Licensing Examination Institute.

**Table 3.** A court case from the 2010s regarding multiple-choice question items of Korean Dental Licensing Examination

Subject of disclosure request	Defendant	Court procedural history	
		Court name & case no. (decision date)	Court’s decision
Korean Dental Licensing Examination	KHPLEI	Seoul Administrative Court 2016Gu-hap60393 Judgment (Oct 20, 2016)	Non-public information
		Seoul High Court 2016Nu69361 Judgment (Jan 23, 2017)	Non-public information
		Supreme Court of Korea 2017Du36625 Judgment (Apr 27, 2017)	Affirm

KHPLEI, Korea Health Personnel Licensing Examination Institute.

**Table 4.** A court case from the 2010s regarding the Clinical Skills Examination of Korean Medical Licensing Examination

Subject of disclosure request	Defendant	Court procedural history	
		Court name & case no. (decision date)	Court’s decision
The 6 CPXs and the 6 OSCEs taken by the examinee-plaintiffs, along with the examinee-plaintiffs’ scores for each CPX and OSCE	KHPLEI	Seoul Administrative Court 2018Gu-hap64139 Decision (Oct 12, 2018)	Public information (affirmed without appeal)
The checklists for each of the 32 OSCEs	KHPLEI	Seoul Administrative Court 2018Gu-hap64139 Decision (Oct 12, 2018)	Non-public information (affirmed without appeal)

CPX, clinical performance examination; OSCE, objective structured clinical examination; KHPLEI, Korea Health Personnel Licensing Examination Institute.

**Table 5.** Court cases from the 2020s regarding multiple-choice questions items prepared by public institutions

Subject of disclosure request	Defendant	Court procedural history	
		Court name & case no. (decision date)	Court’s decision
Grade 9 Civil Service Examination	Governor of Jeju Special Self-Governing Province	Jeju District Court 2020Gu-hap5885 Decision (May 11, 2021)	Non-public information (affirmed without appeal)
Korean Development Bank Examination for Promotion	Chairperson of the Korea Development Bank	Seoul Administrative Court 2021Gu-hap56848 Decision (Jul 15, 2022)	Non-public information (affirmed without appeal)
Veterinary Medical Licensing Examination	Animal and Plant Quarantine Agency Commissioner	Seoul Administrative Court 2023Gu-hap67798 Decision (Jul 11, 2024)	Non-public information
		Seoul High Court 2024Nu54821	Pending on appeal

## Supplementary materials

Supplement 1. Audio recording of the abstract.
